# Crashworthy Performance of Sustainable Filled Structures Using Recycled Beverage Cans and Eco-Friendly Multi-Cell Fillers

**DOI:** 10.3390/polym17030315

**Published:** 2025-01-24

**Authors:** Huijing Gao, Jiangyang Xiang, Junyu Lu, Qianbing Tan, Frédéric Addiego, Yong Peng, Kui Wang

**Affiliations:** 1 Key Laboratory of Traffic Safety on Track of Ministry of Education, School of Traffic & Transportation Engineering, Central South University, Changsha 410075, China; ghjghj@csu.edu.cn (H.G.); xiangjiangyang@csu.edu.cn (J.X.); qianbing.tan@csu.edu.cn (Q.T.); yong_peng@csu.edu.cn (Y.P.); 2 Luxembourg Institute of Science and Technology (LIST), Department Materials Research and Technology (MRT), ZAE Robert Steichen, 5 Rue Bommel, L-4940 Luxembourg, Luxembourg; frederic.addiego@list.lu

**Keywords:** beverage cans, multi-cell fillers, 3D printing, PLA, energy absorption, sustainability

## Abstract

The recycling of resources is an important measure to achieve circular economy and sustainable development. In this paper, a sustainable filled structure was proposed and realized by combining recycled empty beverage cans with eco-friendly multi-cell fillers. Quasi-static axial compressions were carried out to characterize the energy absorption performance and synergistic effect of the filled tubes. Experimental results showed that the crashworthiness of sustainable filled structures varied with both filling densities and materials. With the increase in filling density, the specific energy absorption of the filled tubes presented an upward trend. With the variation in filling materials, the filled tubes exhibited different crashworthiness performances. The PLA multi-cell filled tube could withstand larger external force and exhibited higher SEA values, with a maximum value of 9.64 J/g. The PLAS multi-cell filled tube showed excellent loading stability and lower ULC value, with a minimum value of 10%. These findings provided valuable insights for designing novel sustainable energy absorption structures.

## 1. Introduction

In the field of food packaging, metal cans were extensively utilized due to their light weight, durability, and good sealing performance [1]. According to incomplete statistics, the annual consumption of cans in food packaging worldwide could reach 400 billion. If these cans are not properly managed after use, they can cause persistent environmental pollution and resource consumption. Therefore, proper recycling and reutilization of beverage cans are imperative for environmental preservation, circular economy, and sustainable development [2,3]. As a typical metal thin-walled structure, beverage cans have great potential to dissipate crushing kinetic energy when used as energy absorbers. Palanivelu et al. [4,5] used beverage cans as sacrificial cladding structures to protect civil engineering structures from the air blast load. Ousji et al. [6] investigated the axial crushing response of empty metallic beverage cans under a given blast load and found that the deformation mode of the can followed a non-axisymmetric pattern.

Recently, researchers have tried various methods to improve the energy absorption capacity of empty beverage cans to meet higher requirements, such as applying pressure and foam filling. Yu et al. [7,8] filled beverage cans with compressed air and controlled the internal pressure to create adaptive energy absorbers. The results showed that the mean force of the tubes increased linearly with internal pressure in the diamond mode of the cans. Liu et al. [9] compared the compression resistance of empty beverage cans and foam-filled beverage cans. Experimental results showed that empty beverage cans failed by local buckling, while the foam-filled beverage cans collapsed with a progressive folding process. Chen et al. [10] studied the crashworthiness of foam-filled beverage cans under axial quasi-static crushing, lateral crushing, and low-velocity impact. Wang et al. [11] conducted systematic axial compression tests on composite structures with different sizes of beverage cans and foam filling densities. These studies all demonstrated the effectiveness of combining empty cans with foam to enhance the structural energy absorption capacity.

Filling multi-cell structures was another potential method to enhance crashworthiness [12,13,14,15]. It was reported that inserting some small tubes into a large envelope tube to form an embedded multi-cell structure was an effective way to improve energy absorption. Fu et al. [16] investigated the axial crushing behavior of Nylon and Al/Nylon hybrid tubes. It was found that pure nylon tubes were not desirable energy-absorbing components, but the quadruple-cell Al/Nylon hybrid tubes were satisfactory energy absorbers. Tsang et al. [17] recently proposed a hierarchical tube-in-tube composite structure inspired by the micro-nanostructure of muscle. They introduced seven smaller-diameter tubes into a larger-diameter tube and found that the energy absorption capability was improved significantly. Ying et al. [18] designed a hierarchical hybrid tube, which was composed of two-level components: the external aluminum hollow tube and the internal packing tube. They found that the energy absorption and load-bearing capacity of the hierarchical hybrid tube increased with the number of sub-tubes.

To further enhance environmental protection, biodegradable plastic was selected as the material for multi-cell tubes. Polylactide (PLA) material, as a renewable resource, is one of the most commercially used and successful bioplastics [19]. The biodegradation of PLA produces lactic acid, which is highly biodegradable and biocompatible [20,21]. Due to its environmentally friendly properties, PLA polymer filaments have become the most important thermoplastic source in the three-dimensional (3D) printing field over the last two decades [22,23]. Wu et al. [24] tested the axial crushing of graded honeycombs, which were additively manufactured with PLA. It was found that the specific energy absorption of the optimal bi-graded honeycomb could be 45.6% higher than that of the traditional honeycomb. Rahmatabadi et al. [25] proposed a sustainable 4D printing filament based on PLA. The results revealed that the tensile strength of the filament increased with an increase in the proportion of PLA. Rebelo et al. [26] analyzed the nonlinear response of 3D-printed PLA honeycomb structures under out-of-plane blast loading. The sacrificial cladding solution exhibited a good energy dissipation effect compared with other available materials. These results indicated that PLA-based structures showed feasible energy absorption performance.

In this work, a sustainable filled structure was proposed and realized by combining recycled empty beverage cans with eco-friendly multi-cell fillers. The multi-cell fillers, made with three different densities, were fabricated by fused deposition modeling (FDM) technology using three biodegradable PLA materials. The quasi-static axial compressions were carried out to study the effects of filling density and filling material on the deformation mode and energy absorption performance of the filled tubes. The interaction between the tubes and fillers was further quantitatively investigated. This study aimed to provide a new design scheme for sustainable thin-walled tubes.

## 2. Materials and Processing

### 2.1. Specimen Preparation

**Figure 1:**
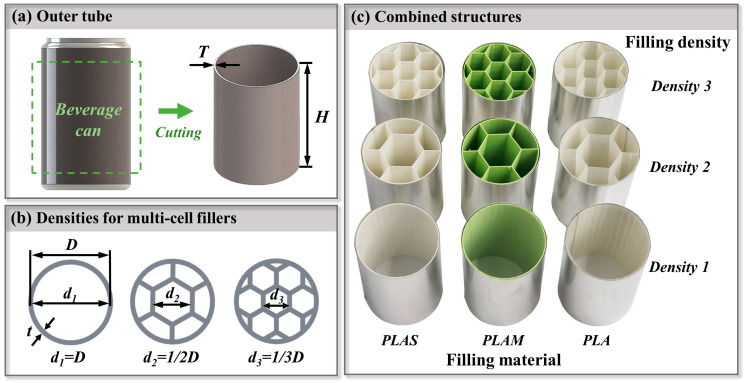
(**a**) Dimensional parameters of repurposed beverage can; (**b**) densities for printed multi-cell fillers; (**c**) combined structures with beverage cans and printed multi-cell fillers.

Steel beverage cans were widely utilized in beverage packaging applications. In this study, a common type of steel beverage cans was purchased from JingCheng (Shenzhen, China). As shown in Figure 1a, the repurposed beverage can had a diameter of 65 mm, a height H of 90 mm, and a thickness T of 0.2 mm. As shown in Figure 1b, the multi-cell fillers had hexagonal cells and were designed with three densities. The literature indicated that hexagonal cells tended to experience stable deformation [27,28]. Based on self-similar design, the geometric dimensions of sub hexagonal cells were 1, 1/2, and 1/3 of the external circle diameter, respectively. All multi-cell fillers had the same thickness t of 0.8 mm and height h of 90 mm. To integrally manufacture these multi-cell structures, a commercial 3D printer (Wiiboox, Nanjing, China) based on the FDM technique was used [29]. Three biodegradable green materials, polylactide (PLA), polylactide matte (PLAM), and polylactide super tough (PLAS), were selected as printing filaments. The printing temperature was 210 °C, and the printing speed was 40 mm/s. Table 1 represents the mechanical properties of the three materials provided by the supplier (eSUN, Shenzhen, China). The data showed that different materials had different strengths and fracture toughness, which would affect the energy absorption characteristics of multi-cell fillers.

Figure 1c shows all filled structures with different filling materials and densities. To investigate the effects of these variations on the energy absorption capacity of filled structures, a full factorial design was implemented to explore all possible combinations. For simplicity, a multi-cell structure made of PLA with density 1 was named PLA1, and the beverage can filled with PLA1 was referred to as PLA1F. By analogy, as the density of multi-cell filler increased, the filled tubes were named PLA1F to PLA3F, PLAM1F to PLAM3F, and PLAS1F to PLAS3F.

### 2.2. Experimental Methods

Quasi-static axial compression tests were conducted to investigate the deformation behaviors and crashworthiness properties of all specimens. A universal material testing machine (MTS E44, Eden Prairie, MN, USA) was used. During compression, the specimen was placed on a rigid bottom platform, and a top platen moved down at a constant speed of 10 mm/min [30]. The specimen was compressed until a total displacement of 65 mm was reached [31]. The loading–displacement data were automatically recorded by a data acquisition system to reflect the compressive response. The whole crushing processes were captured using a digital camera (5D mark IV, Canon, Tokyo, Japan). All the tests were repeated three times, and the average results were reported in this study [32].

### 2.3. Crashworthiness Criteria

To quantitatively evaluate the energy absorption performance, several crashworthiness indicators were used [33]. The energy absorption (EA) is calculated as the integral of the force versus displacement:

(1) $$EA=\int_{0}^{l}F\left(x\right)dx$$

where *l* is the displacement of axial crushing, and *F*(*x*) is the instantaneous crushing force.

The specific energy absorption (SEA) refers to the energy absorption capacity per unit mass of a structure and is one of the most commonly used indicators to measure the crashworthiness performance:

(2) $$SEA=\frac{EA}{m}$$

where *m* is the total mass of each specimen.

The peak crushing force (PCF) refers to the force occurring at the first peak during compression. The mean crushing force (MCF) is defined as the ratio of the energy absorption (EA) to the crushing displacement:

(3) $$MCF=\frac{EA}{l}$$

The crush force efficiency (CFE) can be calculated by dividing the peak crushing force (PCF) by the mean crushing force (MCF). This indicator is used to measure the uniformity of crushing force:

(4) $$CFE=\frac{MCF}{PCF}\times 100\%$$

The undulation of the load-carrying capacity (ULC) is a dimensionless indicator. The smaller the ULC value, the smoother the crushing force oscillations and the better the loading stability:

(5) $$ULC=\frac{\int_{0}^{l}\left|F\left(x\right)-MCF\right|dx}{MCF\times l}\times 100\%$$

## 3. Results and Discussion

### 3.1. The Crushing Behavior of Filled Tubes

**Figure 2:**
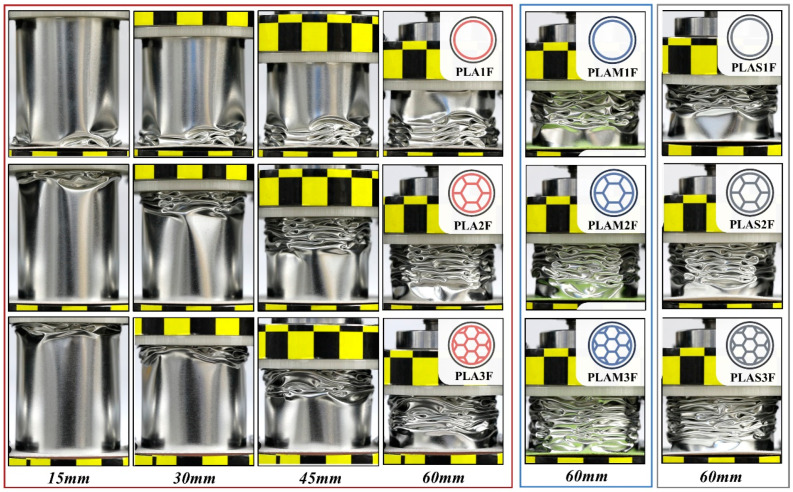
Crushing processes of nine filled tubes.

Figure 2 presents the crushing processes of all filled structures. Initially, the collapse typically began from the moving end of the specimen, which was the end contacting with the upper crosshead, except for PLA1F. During the compression process, the filled structures were crushed layer by layer, and plastic folds were formed with the increased displacement. Finally, all the filled structures exhibited successive plastic deformation and stable progressive collapse, which were considered effective energy absorption modes [34]. To specifically evaluate the influence of different filling materials and densities on the overall folding pattern, a quantitative analysis of the folding number for all filled tubes was subsequently performed.

**Figure 3:**
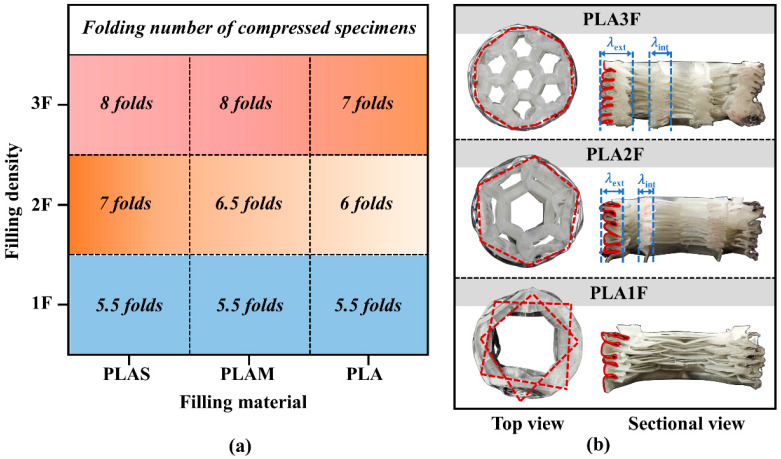
(**a**) Distribution of folding numbers for different filling densities and filling materials; (**b**) the top and sectional views of compressed PLA-filled structures with different filling densities.

Figure 3a shows the folding number of all filled tubes. It was found that the specimens formed more folds as the filling density increased, which was an important mechanism for energy-absorbing structures to dissipate plastic energy [35]. The number of folds also varied with different filling materials, but its effect on the folding pattern was not as great as that of filling density. In particular, the three filled structures with density 1 exhibited the same number of lobes. This was because the relatively low density of the inner tube limited its contribution to the overall energy absorption, and the outer tube played a dominant role in the whole structural deformation.

To further explore the deformation mechanisms of the structures with different filling densities, Figure 3b shows the top view of three compressed PLA-filled beverage cans. For PLA1F, it was found that the collapse mode of the filled tube was similar to the diamond mode of hollow metal circular tubes [36,37]. Four diamond folds were formed in one deformed layer along circumference of the tube. Then, a similar layer developed, which was rotated at a certain angle relative to its adjacent layer. These quadrilateral deformation layers overlapped each other, which presented an asymmetric diamond mode [38]. For PLA2F and PLA3F, the inner multi-cell tube exhibited a progressive collapse mode, while the outer circular tube exhibited an irregular diamond-like mode. The number of polygonal angles formed in folded cans was determined by the configuration of the inner multi-cell tube [39]. These results indicated that as the filling density increased, the influence of the inner multi-cell tubes on the overall deformation became pronounced. For PLAS-filled and PLAM-filled tubes, the failure modes were similar to those of PLA-filled tubes as the filling density changed.

Furthermore, the three PLA-filled specimens were cut along the axial direction, and the sectioned view can be seen in Figure 3b. It was seen that the deformation of the outer beverage can and the inner multi-cell tube was highly consistent. For inner tubes, several cracks were generated at plastic hinge lines. The external wall had obvious and regular folds, and the internal wall exhibited orderly progressive deformation. For the multi-cell tubes of PLA2F and PLA3F, the half-wavelength of internal walls (*λ*_int_) was shorter than that of external walls (*λ*_ext_) due to the different side lengths.

### 3.2. Quantified Evaluation

**Figure 4:**
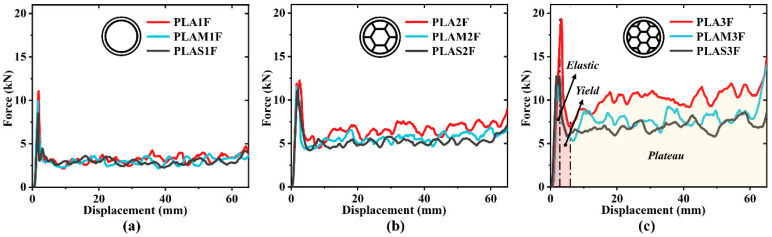
Force–displacement curves of specimens grouped by filling density: (**a**) filled tubes with relatively low filling density; (**b**) filled tubes with middle filling density; (**c**) filled tubes with relatively high filling density.

To characterize the mechanical properties and energy absorption performance of filled structures, all specimens were divided into three groups according to the filling density. The load–displacement curves for each group are plotted in Figure 4. The force responses of all specimens could be divided into elastic, yield, and plateau stages [40]. The force linearly increased to the initial peak in the elastic stage and then dropped sharply due to structural yielding. During the plateau stage, the crushing load fluctuated in a small range around the mean crushing force, corresponding to the progressive folding process of the specimen. It was seen that the duration of both elastic and yield stages was short, and the plateau stage was the main energy absorption phase during the crushing process.

Next, the experimental curves will be discussed in groups. It was found that the force levels of three curves were relatively close, as shown in Figure 4a, while the force levels of three curves exhibited an obvious difference, as shown in Figure 4c. This could be attributed to the competing effects between the outer and inner tubes and the difference in material properties. For filled tubes with low filling density, the outer tube played a dominant role in the overall energy absorption, while the inner tube played a more passive role, as analyzed in Section 3.1. As a result, the force levels of PLA1F, PLAM1F, and PLAS1F were similar across different filling materials. Figure 4c shows that the effect of the inner tube enhanced as the filling density increased. The filling material became a crucial factor. PLA material was characterized by high strength, PLAS material had lower strength, and PLAM material exhibited intermediate strength. Therefore, the force levels of PLA3F, PLAM3F, and PLAS3F were significantly different and gradually decreased.

**Figure 5:**
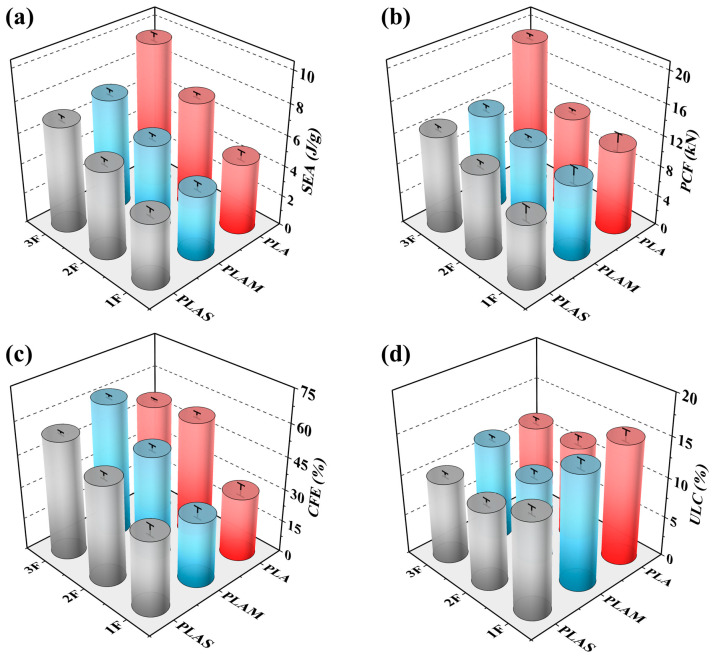
Comparison of crashworthiness indicators of nine filled tubes: (**a**) SEA, (**b**) PCF, (**c**) CFE and (**d**) ULC.

Figure 5 displays quantitative comparisons of crashworthiness indicators. These results show that both filling density and filling material had an impact on overall energy absorption performance. With the increase in filling density, the SEA, PCF, and CFE values of the combined tubes all increased significantly, except for the CFE of PLA3F. As mentioned in Section 3.1, the combined tubes produced more folds and shorter wavelengths with the increase in filling density, which was favorable for energy absorption and structural stability and was reflected in the increase in SEA and CFE values. For the PCF, the structural yield strength increased as the structural density increased, leading to the growth of PCF. For PLA3F tube, since the increase rate of PCF was higher than that of MCF, the CFE of PLA3F decreased compared to that of PLA2F. Therefore, the CFE values of PLA-filled tubes showed a trend of first increasing and then decreasing. In terms of filling materials, different materials endowed the structure with different crashworthiness performance. PLAS exhibited excellent toughness but lower strength, PLA material was characterized by high strength but low toughness, and PLAM properties were somewhere in between. Therefore, PLAS-filled tubes showed smoother crushing force oscillations and lower ULC values. For example, the ULC of PLA3F was 10%, which indicated that the structure had excellent loading stability. The PLA-filled tubes could withstand larger external force and exhibited higher SEA and PCF values. For example, the SEA of PLA3F reached 9.64 J/g. In other studies involving the recycling of waste beverage cans as energy absorbers, the maximum SEA values of these sustainable structures were found to be 8.65 J/g [10], 7.05 J/g [11], and 4.53 J/g [6], which were lower than the value of PLA3F. This comparison demonstrated that our filled structures had great potential in terms of sustainability and energy absorption.

### 3.3. Interaction Between Empty Beverage Cans and Multi-Cell Fillers

**Figure 6:**
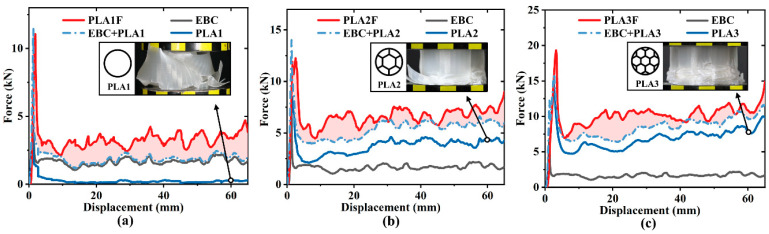
Synergistic effects of filled tubes with different filling densities: (**a**) density 1; (**b**) density 2; (**c**) density 3.

In order to investigate the interaction between empty beverage cans and multi-cell fillers at different filling densities, the compression behavior of three groups of specimens was studied. Figure 6a shows the load–displacement curves of the empty beverage can (EBC), the multi-cell tube made of PLA with density 1 (PLA1), the sum of the empty can and the multi-cell tube (EBC + PLA1), and the corresponding multi-cell-filled tube (PLA1F). The area between curves of EBC + PLA1 and PLA1F represents the synergistic effect of outer can and filler. As shown in Figure 6a–c, the force response levels of three filled tubes were improved owing to the synergistic effect. Notably, this effect in Figure 6a was very remarkable. For PLA1, after exceeding the initial peak, the force fell off rapidly and remained at a low level. From the crushing behavior at the 60 mm displacement, the PLA1 tube developed severe cracks due to brittleness and lost stability, exhibiting a Euler’s buckling mode [40]. In contrast, the EBC had a long loading plateau stage and exhibited relatively stable deformation during compression. As shown in Figure 3b, when PLA1 and EBC were combined, the latter played a dominant role in the overall deformation. Under circumferential constraint of EBC, the failure mode of PLA1 tube changed from Euler’s buckling to asymmetric diamond mode, and large cracked fronds transformed into regular plastic fold lobes. The result indicated that the standalone PLA tube with low density was not suitable for energy absorption due to severe fracture during folding, while this adverse effect could be avoided by adding outer metal cans [41]. The filled tube showed significantly improved energy absorption efficiency.

**Figure 7:**
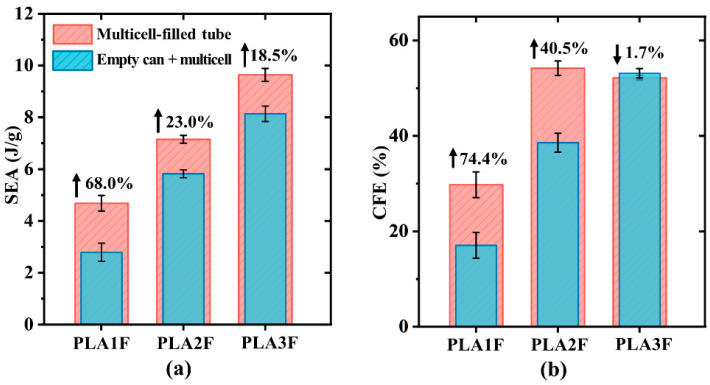
Quantitative analysis of the influence of filling density on synergistic effect on (**a**) SAE and (**b**) CFE.

Figure 7 shows the impact of the synergistic effect on SEA and CFE. The SEA values of three filled tubes were improved compared to the sum of their corresponding components. However, as the filling density increased, this improvement diminished. The synergistic effect in PLA1F was the most significant, resulting in a 68.0% improvement in SEA. For CFE, PLA1F also showed noticeable enhancement, increasing by 74.4%. However, a negative synergistic effect was observed in PLA3F. This was because the inner tube of PLA3F had already exhibited stable progressive cracking failure, and the EBC with lower strength could not further improve the structural stability.

**Figure 8:**
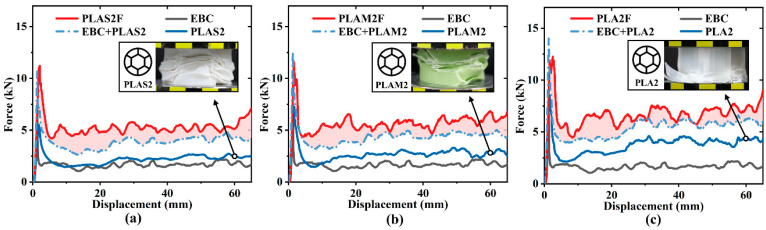
Synergistic effects of filled tubes with different filling materials: (**a**) PLAS, (**b**) PLAM and (**c**) PLA.

To compare the influence of the filling material on synergistic effect, Figure 8 shows the load–displacement curves of three groups of specimens. For three filling materials, their force response levels were higher than the sum of the corresponding components. The main reason was the generation of regular plastic folds and interfacial friction [42,43]. It was found that the multi-cell tubes developed irregular folds when compressed individually, especially for PLAS2 and PLAM2. However, when these tubes were filled into beverage cans, the constraints provided by the outer cans caused the multi-cell tubes to exhibit a more desirable deformation mode with regular plastic fold lobes, which was beneficial to energy absorption. Additionally, the curve fluctuation amplitude of PLA2F in the plateau stage was the largest among three filled tubes. This was because the PLA2F displayed many brittle cracks and fractures, corresponding to the large fluctuations in crushing force.

**Figure 9:**
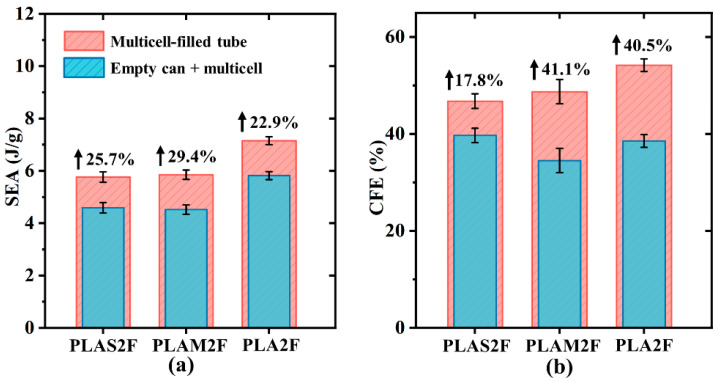
Quantitative analysis of the influence of filling material on synergistic effect on (**a**) SAE and (**b**) CFE.

Figure 9 shows the impact of the synergistic effect on SEA and CFE at different filling materials. The improvement in SEA values was similar for the three filling tubes. For CFE, the improvement in PLAS2F was obviously lower than that of other tubes. This was because the PLAS material had excellent toughness but lower strength. The PLAS2 tube already showed a lower initial peak and good loading uniformity. As a result, the improvement in CFE of PLAS2F was limited.

## 4. Conclusions

In this study, a sustainable filled structure was proposed and realized by combining recycled empty beverage cans with eco-friendly multi-cell fillers. The crushing behaviors and mechanical properties of a series of sustainable filled structures with different filling densities and materials under quasi-static compression were investigated. The interaction between the cans and fillers was further quantitatively analyzed.

Experimental results showed that filling beverage cans with multi-cell structures was an effective approach to enhance crashworthiness. It was clearly observed that the filled tubes formed more folds and shorter wavelengths as the filling density increased, which was favorable for energy absorption. For different filling materials, the structure could be endowed with different crashworthiness performances. The PLA-filled tube could withstand larger external force and exhibited higher SEA and PCF values. The SEA of PLA3F reached 9.64 J/g. The PLAS-filled tube showed excellent loading stability and lower ULC value, and the ULC of PLA3F was 10%. Further experiments showed that the force response levels of filled tubes were higher than the sum of their corresponding components owing to the synergistic effect. The synergistic effect in PLA1F was the most significant, which increased SEA by 68.0% and CFE by 74.4%.

These findings offer valuable insights for the design and regulation of crashworthiness in sustainable energy absorption structures. In our next work, due to the different strain rate and temperature sensitivities of the outer and inner tube materials, the effects of loading rate and temperature on the crushing behaviors of sustainable filled structures can be investigated. Additionally, finite element simulations can be conducted to optimize the structural crashworthiness for specific applications.

## Figures and Tables

**Table 1:** Mechanical properties of the three materials of multi-cell fillers.

Materials	Density (g/cm^3^)	Tensile Strength (MPa)	Elongation at Break (%)
PLA	1.20	65.0	8
PLAM	1.33	42.0	50
PLAS	1.25	34.3	90

## Data Availability

Data are contained within the article.

## References

[1] Lee L., Chen Y., Zheng S., Chang M., Chen J. (2023). Upcycling discarded aluminum cans into nanoporous membranes as an eco-friendly and cost-effective strategy for fabricating polymer nanoarrays and self-cleaning surfaces. ACS Appl. Nano Mater..

[2] Sondh S., Upadhyay D.S., Patel S., Patel R.N. (2024). Strategic approach towards sustainability by promoting circular economy-based municipal solid waste management system—A review. Sustain. Chem. Pharm..

[3] Rabbi M.F., Amin M.B. (2024). Circular economy and sustainable practices in the food industry: A comprehensive bibliometric analysis. Clean. Responsible Consum..

[4] Palanivelu S., Van Paepegem W., Degrieck J., Reymen B., Ndambi J., Vantomme J., Kakogiannis D., Wastiels J., Van Hemelrijck D. (2011). Close-range blast loading on empty recyclable metal beverage cans for use in sacrificial cladding structure. Eng. Struct..

[5] Palanivelu S., Van Paepegem W., Degrieck J., De Pauw S., Vantomme J., Wastiels J., Kakogiannis D., Van Hemelrijck D. (2011). Low velocity axial impact crushing performance of empty recyclable metal beverage cans. Int. J. Impact Eng..

[6] Ousji H., Belkassem B., Pyl L., Vantomme J. (2020). Air-blast loading on empty metallic beverage can used as sacrificial cladding: Experimental, analytical and numerical study. Eng. Struct..

[7] Zhang X.W., Yu T.X. (2009). Energy absorption of pressurized thin-walled circular tubes under axial crushing. Int. J. Mech. Sci..

[8] Hu L.L., Zeng Z.H., Yu T.X. (2016). Axial crushing of pressurized cylindrical tubes. Int. J. Mech. Sci..

[9] Liu C., Qi G., Li P. (2022). Crashworthy characteristics of sustainable thin-walled tubes: A study on recycled beverage cans. Mech. Adv. Mater. Struct..

[10] Chen J., Li E., Liu W., Mao Y., Hou S. (2023). Sustainable composites with ultrahigh energy absorption from beverage cans and polyurethane foam. Compos. Sci. Technol..

[11] Wang Z., Liu Z., Liu Y., Ma W., Zhang Z., Zhao C., Yang C. (2024). On the crush behavior and energy absorption of sustainable beverage cans and their polyurethane foam-filled structures: An experimental study. Materials.

[12] Yao R., Pang T., Zhang B., Fang J., Li Q., Sun G. (2023). On the crashworthiness of thin-walled multi-cell structures and materials: State of the art and prospects. Thin-Walled Struct..

[13] Sun G., Chen D., Zhu G., Li Q. (2022). Lightweight hybrid materials and structures for energy absorption: A state-of-the-art review and outlook. Thin-Walled Struct..

[14] Guo W., Yang L., Xu P., Li S., Yan W., Shen Z., Yao S., Yang C. (2024). Crashworthiness analysis of okra biomimetic corrugated multi-cellular structure. Int. J. Mech. Sci..

[15] Lin P., Zhang Z., Chen Y., Hu D. (2023). Investigation of structural energy absorption performance in 3d-printed polymer (tough 1500 resin) materials with novel multilayer thin-walled sandwich structures inspired by peano space-filling curves. Polymers.

[16] Fu X., Zhang X., Huang Z. (2021). Axial crushing of nylon and al/nylon hybrid tubes by fdm 3d printing. Compos. Struct..

[17] Tsang H.H., Raza S. (2018). Impact energy absorption of bio-inspired tubular sections with structural hierarchy. Compos. Struct..

[18] Ying L., Gao T., Hou W., Dai M., Han X., Jiang D. (2021). On crashing behaviors of bio-inspired hybrid multi-cell al/cfrp hierarchical tube under quasi-static loading: An experimental study. Compos. Struct..

[19] Taib N.A.B., Rahman M.R., Huda D., Kuok K.K., Hamdan S., Bakri M.K.B., Julaihi M.R.M.B., Khan A. (2023). A review on poly lactic acid (pla) as a biodegradable polymer. Polym. Bull..

[20] Akhrib S., Djellali S., Haddaoui N., Karimian D., Carraro M. (2024). Biocomposites and poly (lactic acid) in active packaging: A review of current research and future directions. Polymers.

[21] Tănase M., Portoacă A.I., Diniță A., Brănoiu G., Zamfir F., Sirbu E., Călin C. (2024). Optimizing mechanical properties of recycled 3d-printed pla parts for sustainable packaging solutions using experimental analysis and machine learning. Polymers.

[22] Tümer E.H., Erbil H.Y. (2021). Extrusion-based 3d printing applications of pla composites: A review. Coatings.

[23] Moradi M., Aminzadeh A., Rahmatabadi D., Hakimi A. (2021). Experimental investigation on mechanical characterization of 3d printed pla produced by fused deposition modeling (fdm). Mater. Res. Express.

[24] Wu Y., Sun L., Yang P., Fang J., Li W. (2021). Energy absorption of additively manufactured functionally bi-graded thickness honeycombs subjected to axial loads. Thin-Walled Struct..

[25] Rahmatabadi D., Khajepour M., Bayati A., Mirasadi K., Amin Yousefi M., Shegeft A., Ghasemi I., Baniassadi M., Abrinia K., Bodaghi M. (2024). Advancing sustainable shape memory polymers through 4d printing of polylactic acid-polybutylene adipate terephthalate blends. Eur. Polym. J..

[26] Rebelo H.B., Lecompte D., Cismasiu C., Jonet A., Belkassem B., Maazoun A. (2019). Experimental and numerical investigation on 3d printed pla sacrificial honeycomb cladding. Int. J. Impact Eng..

[27] Alavi Nia A., Parsapour M. (2014). Comparative analysis of energy absorption capacity of simple and multi-cell thin-walled tubes with triangular, square, hexagonal and octagonal sections. Thin-Walled Struct..

[28] Xiong J., Zhang Y., Su L., Zhang F., Wu C. (2022). Experimental and numerical study on mechanical behavior of hybrid multi-cell structures under multi-crushing loads. Thin-Walled Struct..

[29] Xiang J., Cheng P., Wang K., Wu Y., Rao Y., Peng Y. (2024). Interlaminar and translaminar fracture toughness of 3d-printed continuous fiber reinforced composites: A review and prospect. Polym. Compos..

[30] Xiang J., Wang J., Liu Y., Gao H., Tan Q., Wang K., Peng Y. (2025). Effects of undulating printing paths on axial compressive behaviors of 3d-printed continuous fiber-reinforced multi-cell thin-walled structure. Eng. Struct..

[31] Zuo X., Guo C., Chen W., Wang Y., Zhao J., Lv H. (2022). Influence of loading rate and temperature on the energy absorption of 3d-printed polymeric origami tubes under quasi-static loading. Polymers.

[32] Zhu W., Li S., Long H., Dong S., Wang K., Peng Y. (2025). Simulation analysis and optimization of 3d printed continuous carbon fiber reinforced composites. Compos. Struct..

[33] Ha N.S., Lu G. (2020). Thin-walled corrugated structures: A review of crashworthiness designs and energy absorption characteristics. Thin-Walled Struct..

[34] Chen T., Zhang Y., Lin J., Lu Y. (2019). Theoretical analysis and crashworthiness optimization of hybrid multi-cell structures. Thin-Walled Struct..

[35] Liu Y., Tan Q., Lin H., Wang J., Wang K., Peng Y., Yao S. (2023). Integrated design and additive manufacturing of lattice-filled multi-cell tubes. Compos. Sci. Technol..

[36] Pugsley A. (1960). The large-scale crumpling of thin cylindrical columns. Q. J. Mech. Appl. Math..

[37] Faraz M.R., Ahmadi H., Liaghat G., Vahid S., Razmkhah O., Tarafdar A. (2022). Energy absorption assessment of bio-mimicked hybrid al/pp sandwich tube: Experimental and numerical investigation. Thin-Walled Struct..

[38] Guillow S.R., Lu G., Grzebieta R.H. (2001). Quasi-static axial compression of thin-walled circular aluminium tubes. Int. J. Mech. Sci..

[39] Zhang X., Zhang H. (2014). Axial crushing of circular multi-cell columns. Int. J. Impact Eng..

[40] Wang J., Liu Y., Wang K., Yao S., Peng Y., Rao Y., Ahzi S. (2022). Progressive collapse behaviors and mechanisms of 3d printed thin-walled composite structures under multi-conditional loading. Thin-Walled Struct..

[41] Sun G., Li S., Liu Q., Li G., Li Q. (2016). Experimental study on crashworthiness of empty/aluminum foam/honeycomb-filled cfrp tubes. Compos. Struct..

[42] Wang K., Gao H., Wang J., Wang D., Wen W., Peng Y., Yu T. (2024). Modular assembly for multicell structures with designable energy absorption characteristics. Mech. Adv. Mater. Struct..

[43] Nikkhah H., Baroutaji A., Kazancı Z., Arjunan A. (2020). Evaluation of crushing and energy absorption characteristics of bio-inspired nested structures. Thin-Walled Struct..

